# Ghost shrimp *Calliax* de Saint Laurent, 1973 (Decapoda: Axiidea: Callianassidae) in the fossil record: systematics, palaeoecology and palaeobiogeography

**DOI:** 10.11646/zootaxa.3821.1.3

**Published:** 2014-06-19

**Authors:** MATÚŠ HYŽNÝ, ROK GAŠPARIČ

**Affiliations:** 1 Geological-Paleontological Department, Natural History Museum, Vienna, Burgring 7, A-1010 Vienna, Austria. matus.hyzny@nhm-wien.ac.at; 2 Comenius University, Faculty of Natural Sciences, Department of Geology and Palaeontology, Mlynská dolina G1, SVK-842 15 Bratislava, Slovakia; 3 Ljubljanska cesta 4j, 1241 Kamnik, Slovenia. rok.gasparic@gmail.com

**Keywords:** Ghost shrimp, *Calliax michelottii ***comb. nov.**, Oligo-Miocene, systematics, palaeoecology, palaeobiogeography

## Abstract

Ghost shrimps of the family Callianassidae are very common in the fossil record, but mostly as isolated cheliped elements only. The assignment to biologically defined genera, diagnosed on the basis of soft part morphology, is thus rather difficult. In this respect, proxy characters present on chelipeds that are the most durable ghost shrimp remains are needed to ascribe fossil material to extant genera. The genus *Calliax* de Saint Laurent, 1973 has been particularly obscure in this respect. Thorough comparison of extant members of the genus resulted in evaluation of characters present on chelipeds being taxonomically important on the genus level, specifically: 1) rectangular major P1 propodus with two ridges on the base of the fixed finger extending onto manus; 2) major P1 fingers relatively short; and 3) minor P1 chela with dactylus longer than fixed finger and possessing a wide gap between fingers. On this basis, *Callianassa michelottii* A. Milne Edwards, 1860, from the Oligocene and Miocene of Europe is herein reassigned to *Calliax*. Further re-examination of the ghost shrimp fossil record revealed that *C. szobensis* Müller, 1984, from the Middle Miocene of Hungary represents the same animal as *C. michelottii* and they are synomymised herein. The known geographic distribution of *C. michelottii* is expanded by the first confirmed occurrence of the species in Slovakia. All occurrences of *C. michelottii* known to date are reviewed and documented. The presence of *Calliax michelottii* comb. nov. may be considered an indicator of deeper marine settings. Based on the scarce fossil record known to date, *Calliax* has a Tethyan origin; it supposedly migrated westward to establish present day communities in the Caribbean sometime before the Middle Miocene.

## Introduction

Ghost shrimps (Decapoda: Axiidea: Callianassidae) are elongate, soft-bodied, fossorial shrimps with an abdomen distinctly longer than the carapace. They inhabit predominantly shallow intertidal and subtidal marine habitats (or habitats under seawater influence) mainly in the tropics and subtropics (Dworschak 2000, 2005; Dworschak *et al.* 2012). They are known for their sophisticated behaviour, which involves digging complex burrow systems (Griffis & Suchanek 1991; Felder 2001; Dworschak *et al.* 2012), and they can influence the geochemistry of the substrate (Ziebis *et al.* 1996a, b; Felder 2001).

The fossil record of ghost shrimps is very robust and they are present in most associations of Cenozoic decapod crustaceans described so far (Glaessner 1969; Bishop & Williams 2005); however, the generic assignment of their remains is rather difficult because their preservation is often incomplete. In general, there are several different views on the evaluation of taxonomically important characters as exemplified by works of Biffar (1971), Manning & Felder (1991), Ngoc-Ho (2003), Poore (1994, 2008) and Sakai (1999, 2005, 2011). Palaeontological literature usually emphasizes the contribution of Manning & Felder (1991), which treated some characters present on chelipeds as taxonomically important on the genus level.

Neontological and palaeontological practice commonly handles ghost shrimps differently: whereas zoologists usually treat chelipeds as too variable for being used in taxonomy, palaeontologists have to rely on chelipeds, because only rarely they have more than isolated cheliped elements at hand. It has been, however, demonstrated that using statistically robust data can help to resolve the taxonomic problems caused with sexual dimorphism and intraspecific variation, both in neontological (Dworschak 2012) and palaeontological studies (Hyžný 2012; Hyžný & Hudáčková 2012). More comparative research incorporating views from both sides of the discipline are needed to address the generic status of numerous fossil taxa.

Neontological classification of the extant callianassid ghost shrimps relies mostly on the soft part morphology (Mxp3, male Plp1–2, uropods and telson). Although figuring P1 (chelipeds) is a common practice, only limited attention has been paid to the variation in the nature of chelipeds (Dworschak 2006, 2008, 2011, 2012). Moreover, as stated by Dworschak (2012: p. 54), it is important to study specimens of both sexes over a wide size range. This is not always possible to do because larger sample of these fossorial animals is not at hand.

Palaeontological descriptions often lack comparison with extant ghost shrimp genera, partly because a catchall taxon, *Callianassa* Leach, 1814, has been successfully embedded in the minds of palaeontologists and partly because of the rather confused state of neontological classification. Major revisions of Sakai (1999, 2005, 2011) remain questionable at the subfamilial and generic levels (cf. Dworschak 2007; Poore 2008; Dworschak *et al.* 2012). In fact, the basic cheliped morphology of *Callianassa* as commonly recognized by palaeontologists may comprise numerous distinct morphotypes that can be matched to currently recognized extant genera.

The callianassid genus *Calliax* de Saint Laurent, 1973, has rather complex taxonomic history and only limited attention has been paid to it when evaluating the ghost shrimp fossil remains. Little fossil material has ever been assigned to the genus (Swen *et al.* 2001; Schweitzer *et al.* 2003; Charbonnier *et al.* 2013), although the present study shows that its fossil record is not as poor as previously thought (Schweitzer *et al.* 2003). This, of course, owes much to the preservational bias toward isolated cheliped elements, whereas features of the carapace, maxillipeds, eyes, pleopods, uropods and telson, which are used to assign extant species to the genus, are very rarely if at all preserved in the fossil record. It has been argued that at least for some genera the morphology of the major propodus can be diagnostic on the genus level (Hyžný 2012; Hyžný & Müller 2012). In this respect, the revision of the ghost shrimp fossil record is ongoing (Hyžný & Müller 2010, 2012; Hyžný 2012; Hyžný & Hudáčková 2012; Hyžný & Karasawa 2012; Hyžný *et al.* 2013). This paper is intended to be a part of the revision and its goal is to discuss in detail assignment of fossil taxa to *Calliax* based on the case study of *Callianassa michelottii* A. Milne Edwards, 1860, from the Oligocene and Miocene of Europe.

### Localities and geological settings

**FIGURE 1:**
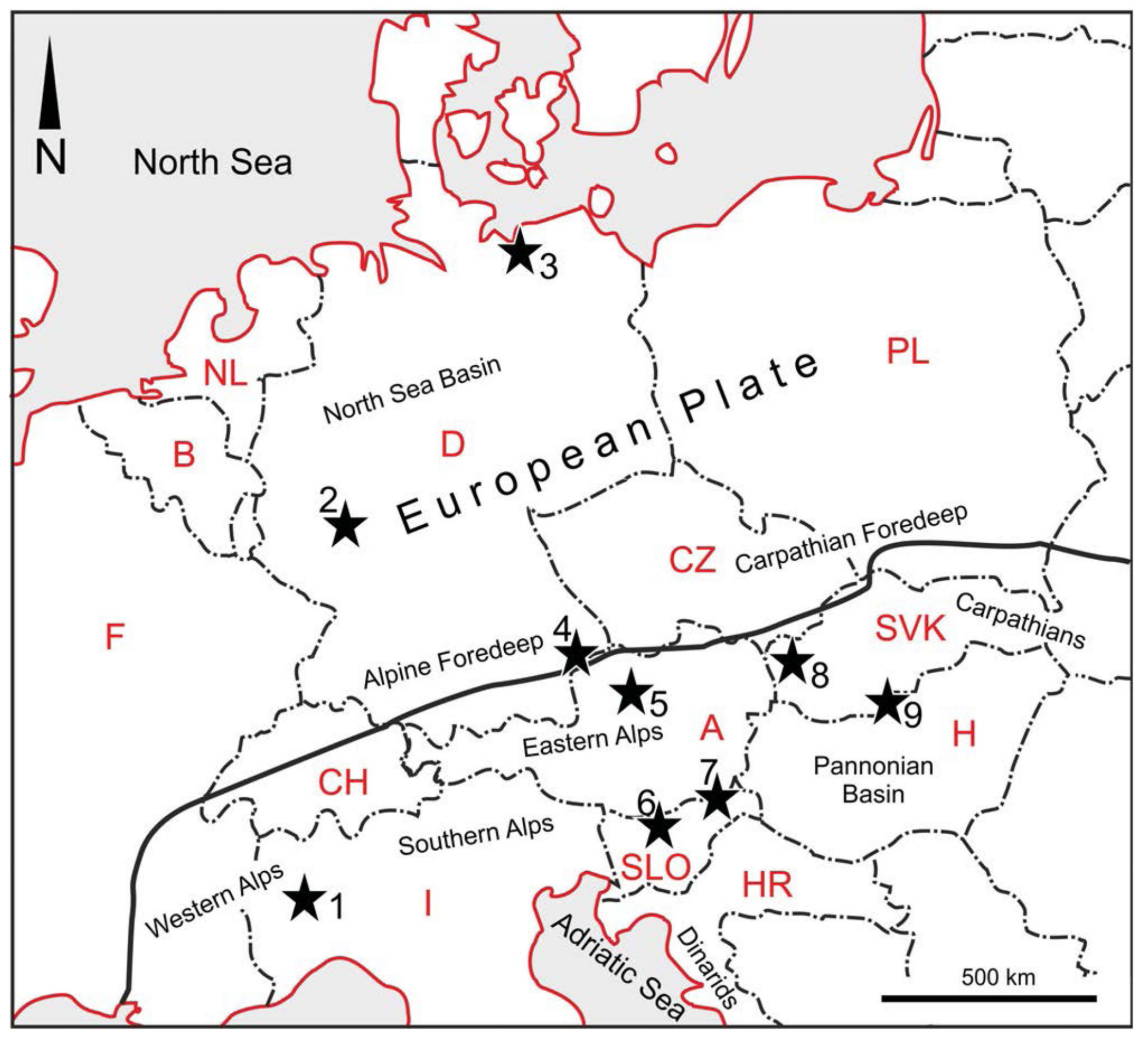
Occurrences of *Calliax michelottii* (A. Milne Edwards, 1860) comb. nov. 1=type locality at Superga at Turin (Italy); 2=Flörsheim, Offenbach bei Frankfurt (Germany); 3=Sternberg, Pinnow bei Schwerin, Kobrow (Germany); 4=Neuhofen (Germany); 5=Pucking (Austria); 6=Kamnik (Slovenia); 7=Jarenina (Slovenia); 8=Rohožník (Slovakia); 9=Želiezovce (Slovakia), Szob (Hungary). The map outline is adopted from Rasser & Harzhauser (2008: fig. 17.2). A thick line marks the border between the European Plate and the Alpine-Carpathian orogenic system. Abbreviations correspond to official country code plates.

Personally studied fossil material consists of specimens from two different areas (Fig. 1):

1) NW part of Mecklenburg-Vorpommern (Germany). At several places the calcareous and siderite sandstone of Oligocene (Chattian) age is exposed. These strata are known as “Sternberger Gestein” and are extremely fossiliferous (e.g. Janke 1993). Specimens of *Callianassa michelottii* studied herein originate from exposures at Sternberg and Pinnow bei Schwerin.
2) Neogene basins of the former Central Paratethys Sea. The presence of *C. michelottii* has been confirmed at several Oligo-Miocene (Chattian–Serravallian) localities of Austria, Germany, Slovakia, Hungary and Slovenia, namely:
  - North Alpine Foreland Basin (Austria)—Pucking locality was a temporary outcrop in the vicinity of Pucking in Upper Austria. Laminated sandy and silty clays of the Ebelsberg Formation (Krenmayr & Schnabel 2006) were exposed here. They are part of the upper Egerian stage (=Aquitanian) and correspond to the nannoplankton zone NN2 (Gregorová *et al.* 2009; Grunert *et al.* 2010). Various aspects of the fossil fauna of the locality were discussed by Gregorová *et al.* (2009), Grunert *et al.* (2010), Harzhauser *et al.* (2011) and Harzhauser & Schlögl (2012).
  - North Alpine Foreland Basin (Germany)—At Neuhofen bei Tettenweis the type locality of the Neuhof Beds (Neuhofener Schichten) is situated. They are composed of clayey to fine sandy marls (Doppler *et al.* 2005). The Neuhof Beds in the Eastern Mollasse are the equivalent of the Kalkofen Formation in the Western Molasse, and their age is Lower Ottnangian (Burdigallian) (Heckeberg *et al.* 2010).
  - Vienna Basin (Slovakia)—At the Rohožník locality marine sediments of the Studienka Formation (Vass 2002) were exposed for clay mining. The age has been identified as the Middle Badenian–Sarmatian (Langhian–Serravallian) (Fuksi *et al.* 2011). Fossil association consists of 70 species of Foraminifera, 26 mollusc genera, and several species of decapod crustaceans (Fuksi *et al.* 2011).
  - Great Hungarian Plain (Hungary)—At Szob, sandstone, sand, and tuffaceous silt were exposed (Müller 1984). The locality yielded only a few fragmented remains, *Callianassa szobensis* Müller 1984 (=*Callianassa michelottii*) among them. The age is Lower Badenian (NN5 Biozone; Langhian) (Nagymarosi *in* Müller 1984).
  - Styrian Basin (Slovenia)—at locality Jarenina (Jahring) Helvetian (~Karpatian, late Burdigalian) “Schlier” facies is exposed. Glaessner (1928) reported several isolated chelae of *Callianassa michelottii*. Another species, *Callianassa jahringensis* Glaessner, 1928, from the same locality differs significantly from *C. michelottii* and belongs to a different genus (Hyžný in prep.).
  - Sava folds Basin (Slovenia)—at the locality Košiše, ca. 3 km NE of Kamnik (Stein in Oberkrain) in central Slovenia grey to yellowish sandstones of the Laško Formation are exposed. The age of the beds have been estimated to be Langhian/Serravallian (Badenian). The exposure at Košiše is the type locality of *Cancer carniolicus* Bittner, 1884 (now treated as *Tasadia* Müller *in* Janssen & Müller, 1984), which is the most common element of the decapod association here (Mikuž & Pavšič 2003). *Callianassa michelottii* is relatively common also. Molluscs and shark teeth of the Laško Formation were briefly reported by Žalohar & Zevnik (2006) and Žalohar *et al.* (2006), respectively.

For the palaeogeographic concept of the Central Paratethys during the Miocene a reference is made to Rögl (1998, 1999), Harzhauser *et al.* (2002) and Harzhauser & Piller (2007). The current status of the Miocene Central Paratethys stratigraphy was summarized by Piller *et al.* (2007). The timing of the Badenian stage was recently revised by Hohenegger *et al.* (2014).

## Material and methods

The fossil material 1) comes from older collections (samples from Germany, Austria, Hungary and partly Slovenia and Slovakia), 2) was donated by private collectors to the authors for study (sample from Slovakia), 3) or was collected by the junior author (sample from Slovenia). For more details see the Systematics chapter and acknowledgements.

**FIGURE 2:**
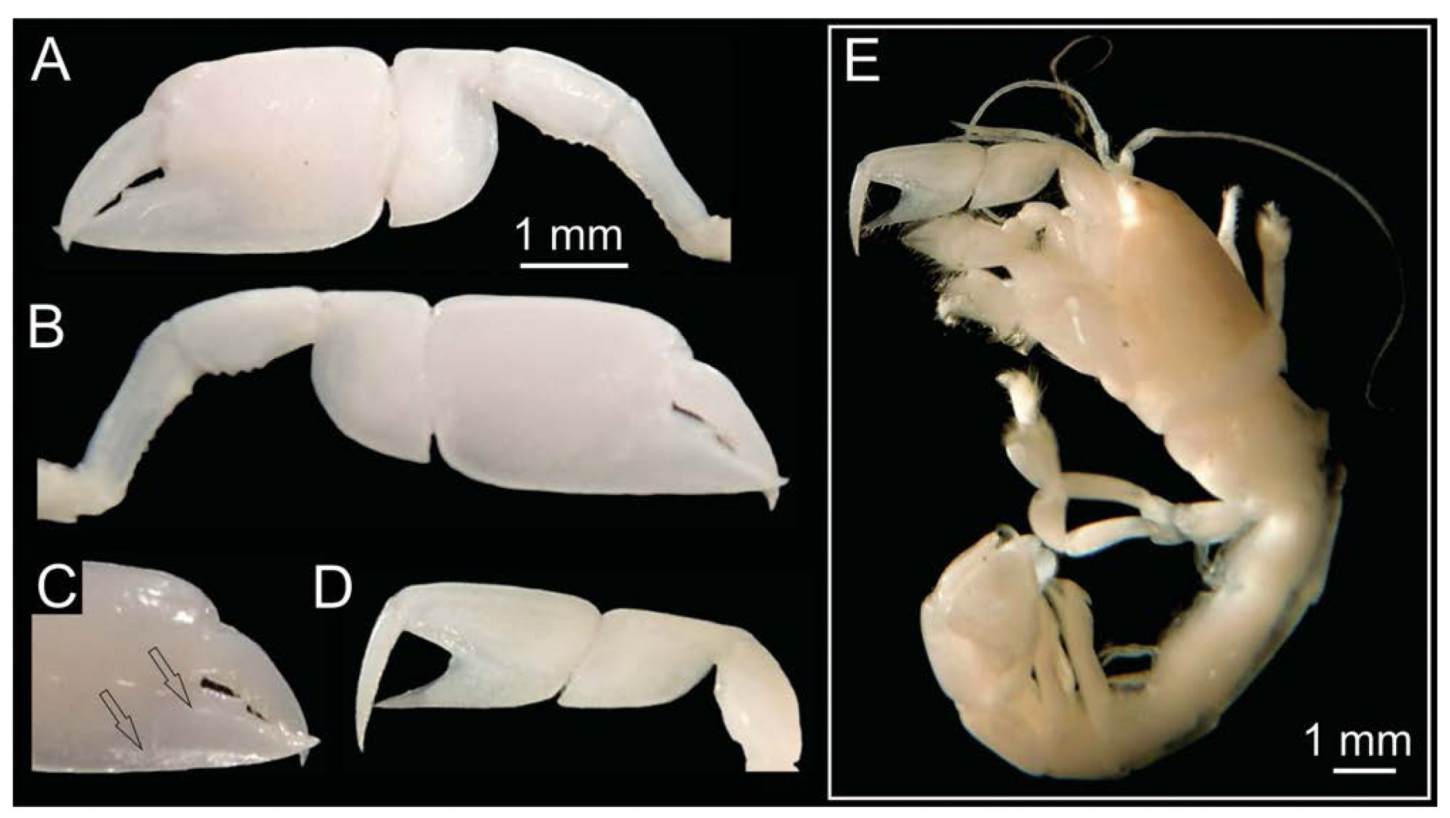
*Calliax* cf. *C. lobata*, NHMW 25511, female, Anastasya mud volcano, 457 m, Gulf of Cádiz, Spain: A, inner view of major P1; B, outer view of major P1; C, ridges (arrows) on the fixed finger under low-angled light; D; outer view of minor P1; E, entire animal (major P1 is detached).

**FIGURE 3:**
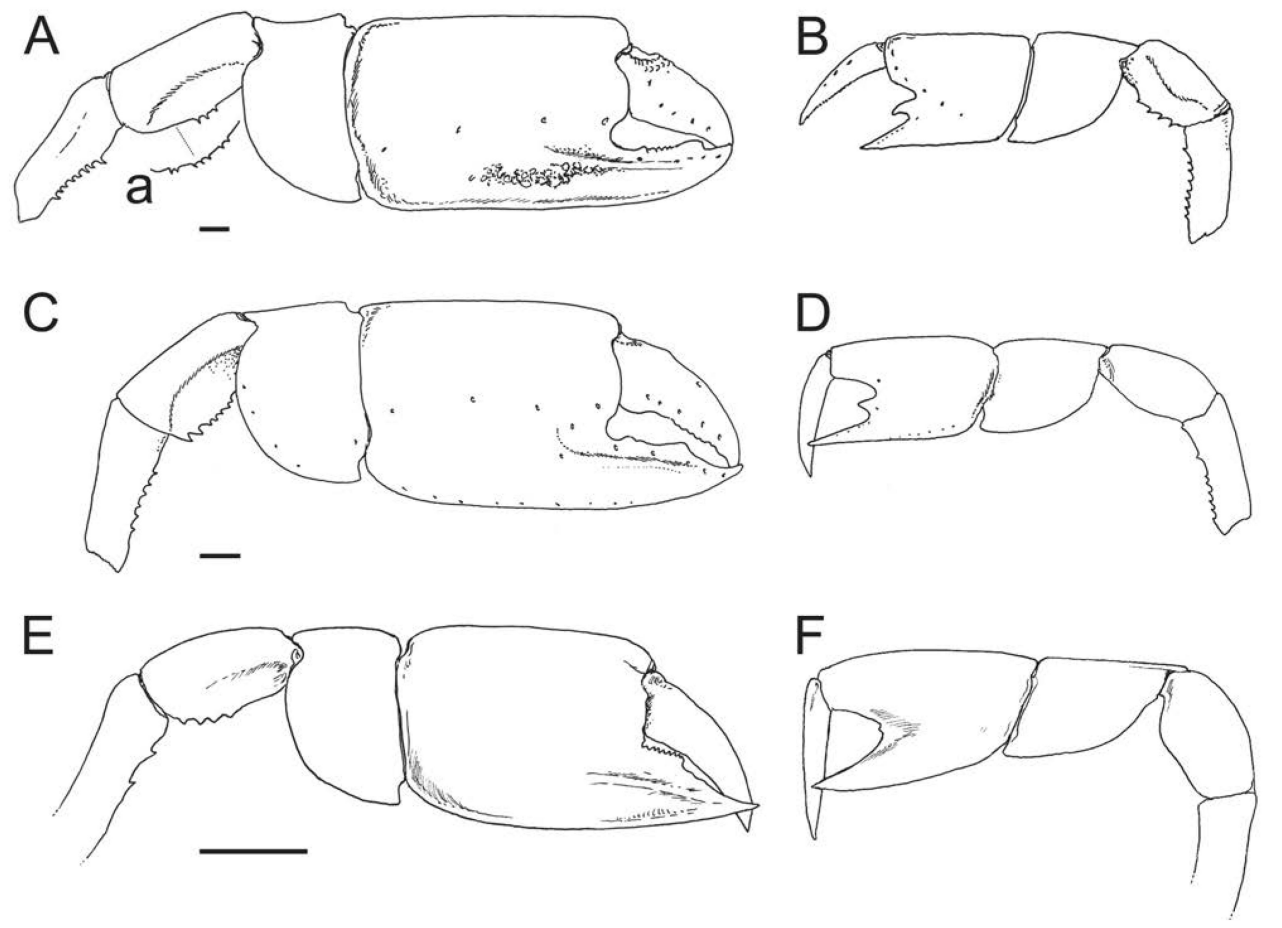
Extant *Calliax* species: A–B, *Calliax doerjesti* Sakai, 1999, note the discrepancy in figuring lower margin of major P1 merus in “A” and “a” (redrawn after Sakai 1999: figs 28–29); C–D, *Calliax lobata* (de Gaillande & Lagardère, 1966) (redrawn after Ngoc-Ho 2003: figs 17D–E); E–F, *Calliax* cf. *C. lobata*, line drawings of NHMW 25511 (Fig. 2). Left column=major chelae (outer surface), right column=minor chelae (outer surface). Setae are omitted. From two ridges at the base of the fixed finger only the more developed one is depicted in the sketches (see text for details). Scale bar equals 1 mm.

For comparative purposes, extant material has also been examined. The extant material used for this study comprises *Calliax* cf. *C. lobata*: NHMW 25511, female, Anastasya mud volcano, 457 m, Gulf of Cádiz, Spain (Figs 2, 3E–F).

Fossil material was photographed either dry and uncoated or coated with ammonium choride sublimate. The line drawings of extant material have been made using camera lucida.

Studied material is deposited in the following institutions: Geologische Bundesanstalt, Vienna, Austria (GBA); Department of Geology and Palaeontology, Comenius University, Bratislava, Slovakia (KGP MH); Universalmuseum Joanneum, Graz, Austria (UMJGP); Hungarian Natural History Museum, Budapest, Hungary (M); Muséum National d’Histoire Naturelle, Paris, France (MNHN); Naturhistorisches Museum Wien, Austria (NHMW); and Slovenian Museum of Natural History, Ljubljana, Slovenia (RGA/SMNH).

*Abbreviations used in text*. A1, antennule; A2, antenna; Mxp1–3, maxillipeds 1–3; P1–5, pereiopods 1–5; Plp1–5, pleopods 1–5.

### Systematics

### Order Decapoda Latreille, 1802

### Infraorder Axiidea de Saint Laurent, 1979

### Family Callianassidae Dana, 1852

#### Remarks

Ghost shrimps are usually strongly heterochelous. Only a few taxa have subequal chelipeds, such as eucalliacine genera *Calliaxina* Ngoc-Ho, 2003; and *Eucalliax* Manning & Felder, 1991. The chelipeds (P1) of callianassid shrimps are laterally flattened and are subject of intraspecific variation as well as sexual dimorphism (or even polymorphism) (e.g. Manning & Felder 1986; Felder & Lovett 1989; Schweitzer Hopkins & Feldmann 1997; Swen *et al.* 2001; Mourik *et al.* 2005; East 2006). Manning & Felder (1991) turned attention to the characters on chelipeds, although they discussed extant American taxa only. The taxonomic importance of the chelipeds in systematics of callianassid genera was emphasized also by Ngoc-Ho (2003) when comparing genera within the subfamily Eucalliacinae. Such studies are considered of great importance for palaeontologists working with incompletely preserved individuals.

### Subfamily Eucalliacinae Manning & Felder, 1991

#### Remarks

Discussion of the fossil record of the subfamily was provided by Hyžný & Hudáčková (2012) and Hyžný (2012). Sakai (2011) reconsidered Eucalliacinae and elevated it to familial level. Moreover, he added the monogeneric Calliapaguropinae Sakai, 1999, to the Eucalliacidae. Following De Grave *et al.* (2009), the Eucalliacinae is treated here as subfamily. For a listing of all extant species of Eucalliacinae with the taxonomic history of their generic assignment, reference is made to Hyžný (2012: table 1). Since then, Sakai & Türkay (2014) erected another genus and species, *Calliaxiopsis madagassa*.

### Genus *Calliax* de Saint Laurent, 1973

*Type species.—Callianassa* (*Callichirus*) *lobata* de Gaillande & Lagardère, 1966.

*Extant species included*.—Three species (including one referred species but not formally named): *Calliax doerjesti* Sakai, 1999 (Figs 3A–B); *Calliax lobata* (de Gaillande & Lagardère, 1966) (Figs 3C–D); *Calliax* sp. *sensu* Taviani *et al.* (2013).

*Fossil species included*.–*Calliax michelottii* (A. Milne Edwards, 1860) comb. nov. More fossil occurrences in open nomenclature are recognized (see Table 1).

#### Diagnosis

Carapace lacking dorsal oval; rostrum short, with blunt tip, rostral spine absent. Pleonal segment 2 longest, no lateral tufts of setae on segments 3–5. Telson slightly wider than long, lateral margin curved, posterior margin straight or slightly convex. Eyestalk about twice as long as wide, slightly flattened dorso-ventrally; cornea small, weakly pigmented. A1 peduncle shorter than that of A2. Mxp1 epipod tapering anteriorly. Mxp2 with small, leaf-like epipod. Mxp3 subpediform (*sensu* Ngoc-Ho 2003), propodus and dactylus rounded, exopod absent. P1 unequal, dissimilar. Major P1 propodus rectangular, usually longer than high, fixed finger shorter than manus, with a double ridge accompanied by a furrow extending onto manus and parallel to the lower margin of propodus. Fixed finger as long as dactylus in major P1, shorter than dactylus in minor P1, with wide proximal gap and large triangular proximal tooth on cutting edge. Major P1 carpus shorter than high, distinctly shorter than propodus. Major P1 merus longer than high, keeled, lower margin armed with small spines. P3 with small proximal heel on propodus, P5 subchelate. Paired arthrobranch on Mxp3 and P1–4. Male and female Plp1 uniramous male and female Plp2 biramous, all lacking *appendix interna*, male Plp2 with *appendix masculina* overreaching endopod. Plp3–5 biramous, foliaceous, *appendix interna* finger-like in both sexes. Uropodal endopod and exopod slightly longer than telson, with rounded posterior margin; exopod with dorsal plate terminating in short distal setal row [emended from Ngoc-Ho (2003: 489) with characters on major P1].

#### Remarks on the taxonomy

*Calliax* has a complex taxonomic history. The genus was erected by de Saint Laurent (1973) with *Callianassa lobata* de Gaillande & Lagardère, 1966, as the type species. Since then the concept of the genus has been changed several times (cf. Manning & Felder 1991; Sakai 1999, 2005, 2011; Ngoc-Ho 2003; Hyžný 2012; see also Dworschak 2007). Here the view of Ngoc-Ho (2003) and Sakai (2011) is adopted, and thus, only two formally described extant species are recognized. Discussion on distinguishing *Calliax* from related taxa based on soft-part morphology was provided by Ngoc-Ho (2003) and will not be repeated here.

When dealing with chelipeds the two known extant species of *Calliax* can be characterized by unequal and dissimilar chelae, from which the minor one has “fixed finger shorter than and separated from the dactylus by a wide gap, bearing a large triangular proximal tooth (Ngoc-Ho 2003: 490)”. This morphology approaches a subchelate cheliped state. Comparison of the illustrated major P1 propodus of *C. lobata* (de Gaillande & Lagardère 1966: fig. 2a; de Saint Laurent & Božić 1976: fig. 23; Ngoc-Ho 2003: fig. 17D), *C. doerjesti* (Sakai 1999: figs. 28, 29b) and *Calliax* sp. (Taviani *et al.* 2013: fig. 8) clearly shows consistency in its general shape, i.e. propodus is rectangular and usually longer than high and on its lateral surface the fixed finger possesses two ridges accompanied by furrow (or furrows) parallel to the lower margin of propodus and extending onto manus. The ridges are visible especially when viewing under low angle light (Fig. 2C). There are several distinct setal pores (accompanied by tubercles) arranged obliquely across the lateral surface of propodus. In *Calliax* the major P1 carpus is always much shorter than manus, with rounded proximo-lower margin (Fig. 3). The merus is longer than high with a distinct meral keel and its lower margin is usually armed with small spines. The above mentioned combination of the characters of minor chela and major propodus, carpus and merus is unique for *Calliax*; thus, the genus can be identified on the basis of chelipeds alone.

**FIGURE 4:**
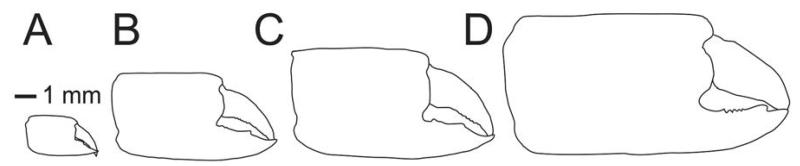
Comparison of figured major chela of *Calliax*: A, *Calliax* cf. *C. lobata* (NHMW 25511); B, *Calliax lobata* (redrawn after de Saint Laurent & Božić 1976: fig. 23a); C, *Calliax lobata* (redrawn after Ngoc-Ho 2003: fig. 17D); D, *Calliax doerjesti* (redrawn after Sakai 1999: fig. 29b). All figures are to scale.

The number of spines on the lower margin of P1 merus may vary between respective members of *Calliax* and may help in distinguishing taxa at the species level, although possible variation has not been studied in detail yet. Regarding the number of meral spines, there are discrepancies in the literature. Sakai (1999: 114) in the description of *C. doerjesti* mentioned that the lower margin of the merus was “armed with three interspaced denticles”. One of the figures (Sakai 1999: fig. 29b) indeed shows three small spines, however, in the other one (Sakai 1999: fig. 28) depicting the same specimen (holotype) the merus is armed with seven spines (Fig. 3a). Ngoc-Ho (2003: fig. 17D; note that the published figure depicts the right major chela, whereas the caption refers to it as the left one) figured the holotype (male) of *C. lobata* with seven spines on the merus and de Saint Laurent & Božić (1976: fig. 23a) figured a female specimen of *C. lobata* also with seven spines. *Calliax* cf. *C. lobata*, examined and figured herein (Figs 2, 3E–F), possesses only four blunt spines, presumably mirroring its small size (Fig. 4).

#### Remarks on the fossil record

Articulated chelipeds are relatively sparse in the fossil state and often only isolated propodi are at hand. In this respect, the major P1 propodus of *Calliax* is distinct enough to be differentiated from all other ghost shrimp genera. It must be stressed, however, that the more cheliped elements are found, the more secure assignment at the genus level can be provided.

**FIGURE 5:**
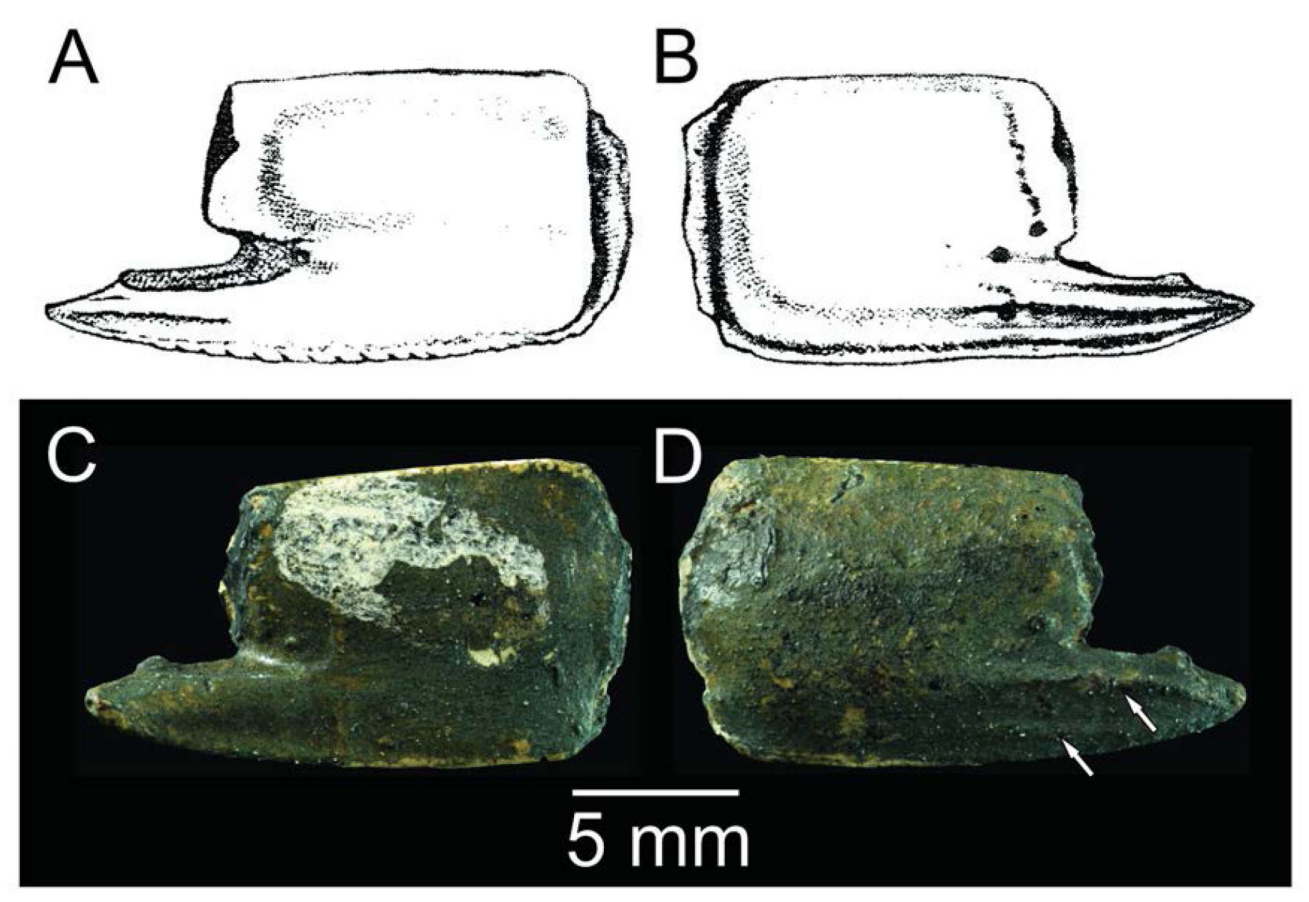
*Callianassa michelottii* A. Milne Edwards, 1860: holotype (MNHN-F-B32690): A–B, refigured from A. Milne Edwards (1860: pl. 14, fig. 3); C–D, photo of the holotype. A, C=inner surface; B, D=outer surface. Arrows indicate ridges. Photo by Lilian Cazes (MNHN).

**FIGURE 6:**
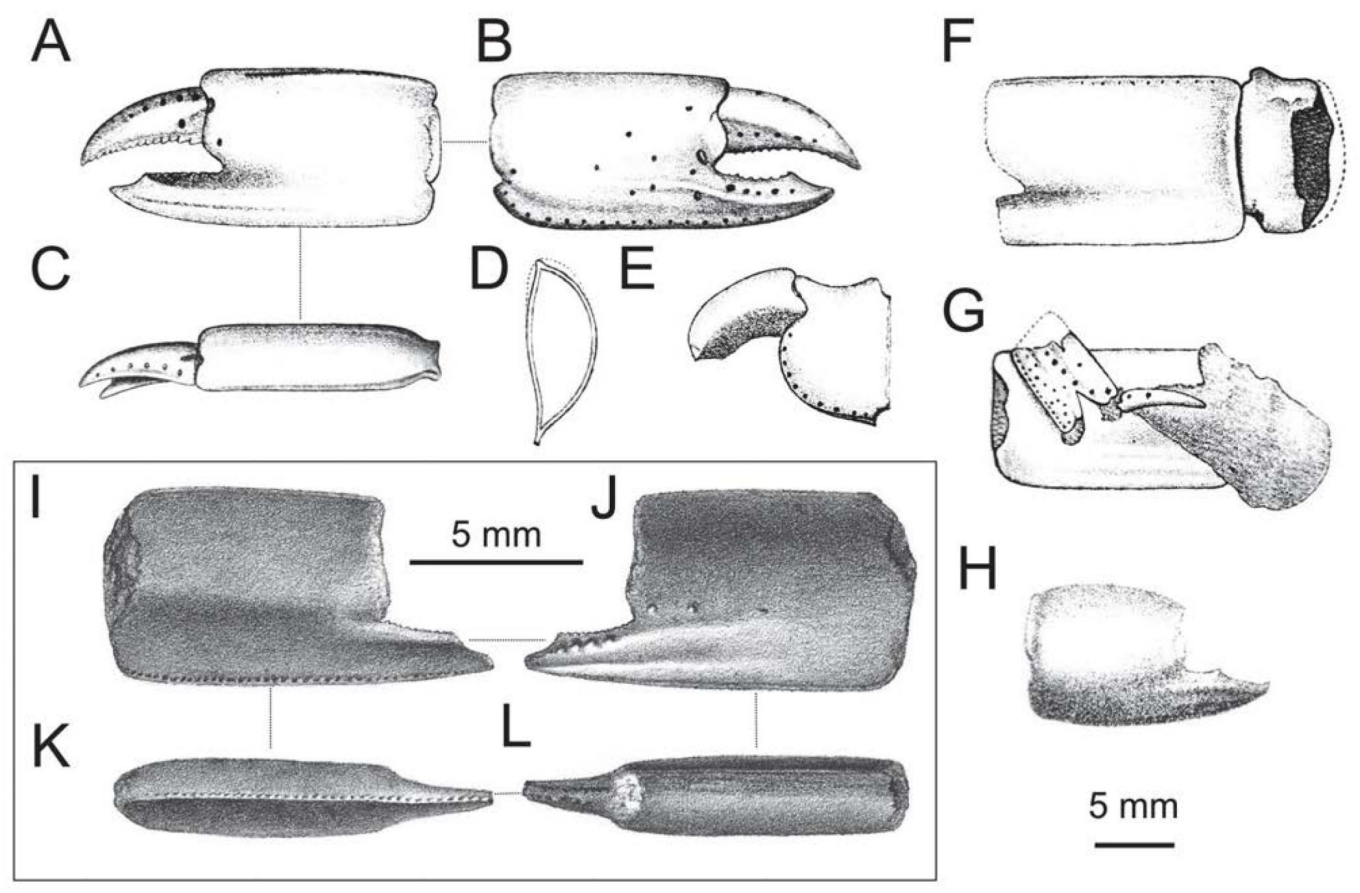
*Calliax michelottii* (A. Milne Edwards, 1860) comb. nov., occurrences published previously. A–G: Oligocene of the Mainz Basin, Flörsheim and Offenbach at Frankfurt (Germany). A–C, right major P1 propodus articulated with dactylus from inner, outer and upper view, respectively; D, cross section of major P1 propodus; E, right major P1 merus articulated with carpus; F, right major P1 propodus articulated with carpus in inner view; G, left major P1 propodus associated with right minor P1 chela (propodus articulated with dactylus). H: Miocene of Sardinia (Italy), the assignment to *C. michelottii* comb. nov. is questionable. I–L: Oligocene of “Sternberger Gestein”, Kobrow (Germany), left major P1 propodus in inner (I), outer (J), lower (K) and upper view (L). A–G are refigured from Fritsch (1871), H is refigured from Lőrenthey (1907), I–L are refigured from Noetling (1886). A–H are to scale, scale bar equals 5 mm.

**FIGURE 7:**
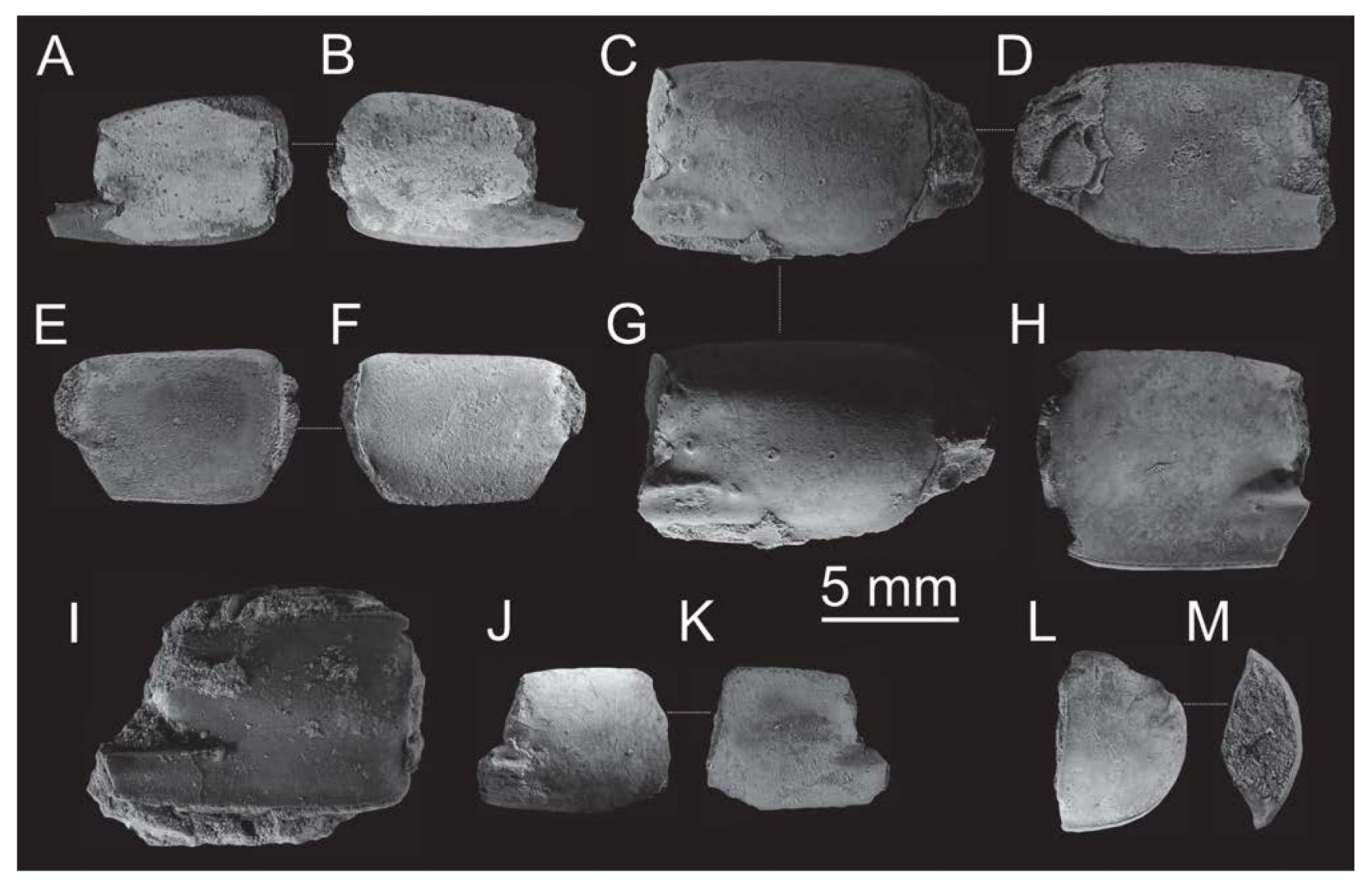
*Calliax michelottii* (A. Milne Edwards, 1860) comb. nov. from the Oligocene of the European plate: isolated propodi (A–K) and carpus (L–M). A–B, NHMW 1874/0029/1154 a; C, D, G, NHMW 1874/0029/1154 a, G shows the specimen under a low-angle light to show the ridges on the fixed finger; E–F, NHMW 1874/0029/1154 c; H, NHMW 1874/0029/1154 d; I, NHMW 1874/0029/1155; J–K, NHMW 1874/0029/1154 e; L–M, NHMW 1874/0029/1154 f. Specimens in A–H, J–M are from Sternberg (Germany), specimen in I is from Pinnow bei Schwerin (Germany). All specimens are to scale and were covered with ammonium chloride prior to photography. Scale bar equals 5 mm.

**FIGURE 8:**
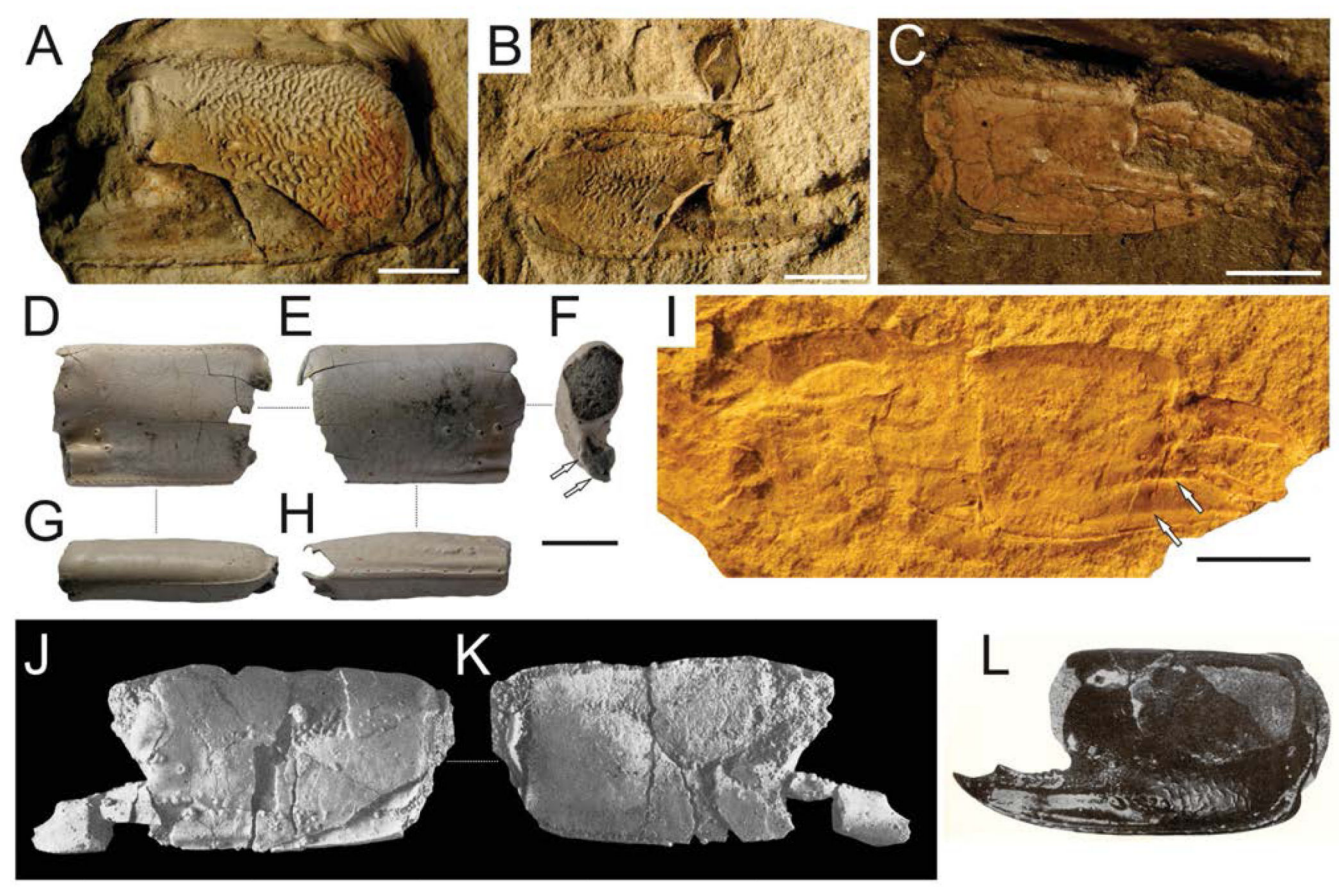
*Calliax michelottii* (A. Milne Edwards, 1860) comb. nov. from the Alpine-Carpathian orogenic system. A–B, UMJGP 77873–77874, isolated propodi from Jarenina (Slovenia); C, NHMW 2003/0026/0913, right propodus articulated with dactylus from Pucking (Austria); D–H, KGP-MH RO-001, fragmentary right propodus from Rohožník (Slovakia), note large setal pores on the outer lateral surface (E), arrows (in F) indicate ridges; I, NHMW 2010/0089/0001, counterpart of the major left cheliped from Neuhofen bei Tettenweis (Germany), arrows indicate ridges; J–K, M.2004.158.1, holotype of *Callianassa szobensis* Müller, 1984 from Szob (Hungary), refigured from Müller (1984: pl. 7, figs 3–4); L, left major propodus from Želiezovce (Slovakia), refigured from Špinar *et al.* (1965: fig. X-184). Scale bar equals 5 mm.

**FIGURE 9:**
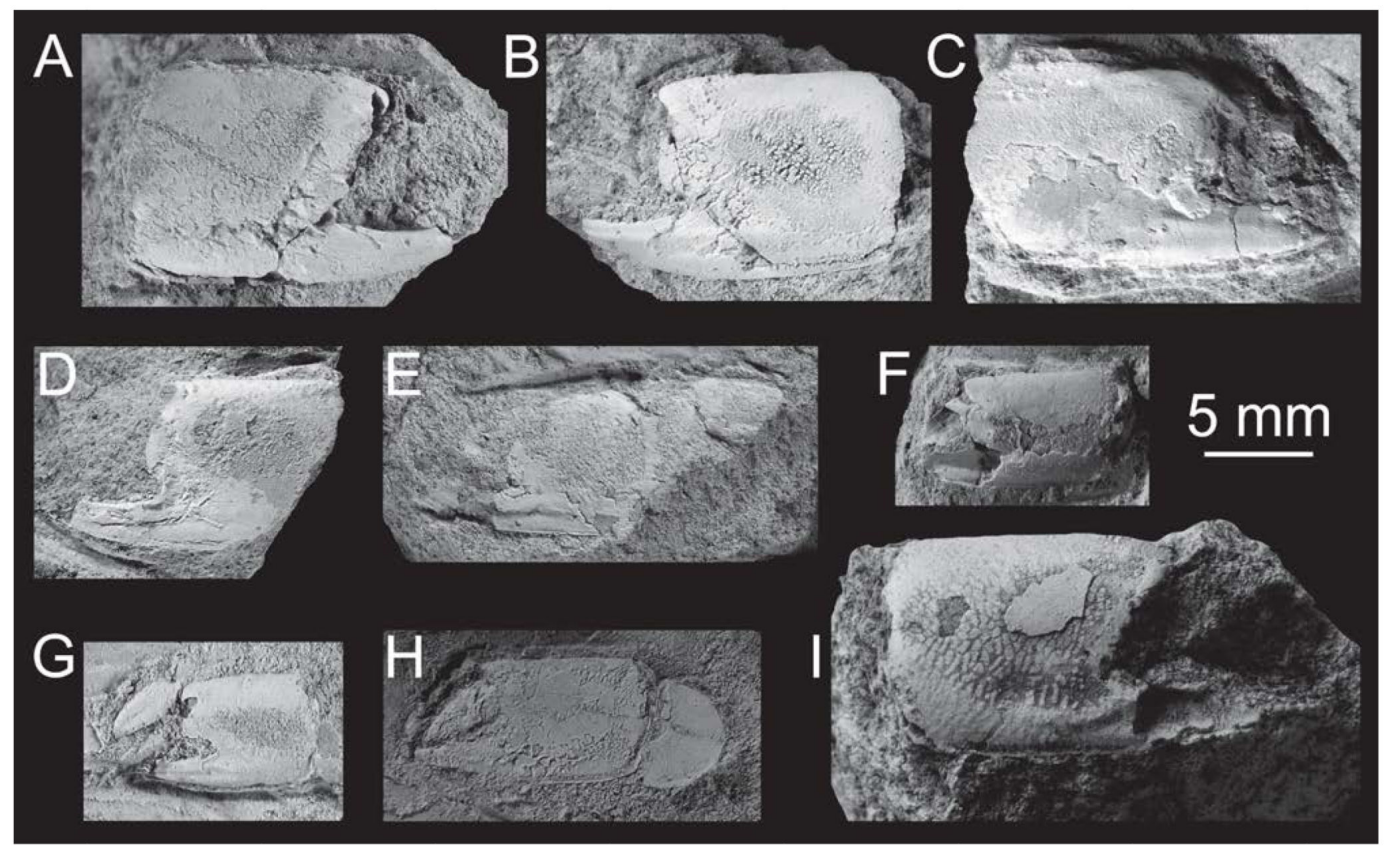
*Calliax michelottii* (A. Milne Edwards, 1860) comb. nov. from the Middle Miocene of Kamnik, Slovenia. A, RGA/SMNH 0754, right propodus; B, RGA/SMNH 0779, left propodus; C, RGA/SMNH 0864, right propodus; D, RGA/SMNH 0773, left propodus; E, RGA/SMNH 1191, left major chela consisting of propodus, carpus and merus; F, RGA/SMNH 1075, left propodus articulated with dactylus; G, RGA/SMNH 1187, left propodus articulated with dactylus; H, RGA/SMNH 1069, left major chela consisting of dactylus, propodus and carpus; I , GBA 2009/014/0027, right propodus. All specimens are to scale and were covered with ammonium chloride prior to photography. Scale bar equals 5 mm.

**FIGURE 10:**
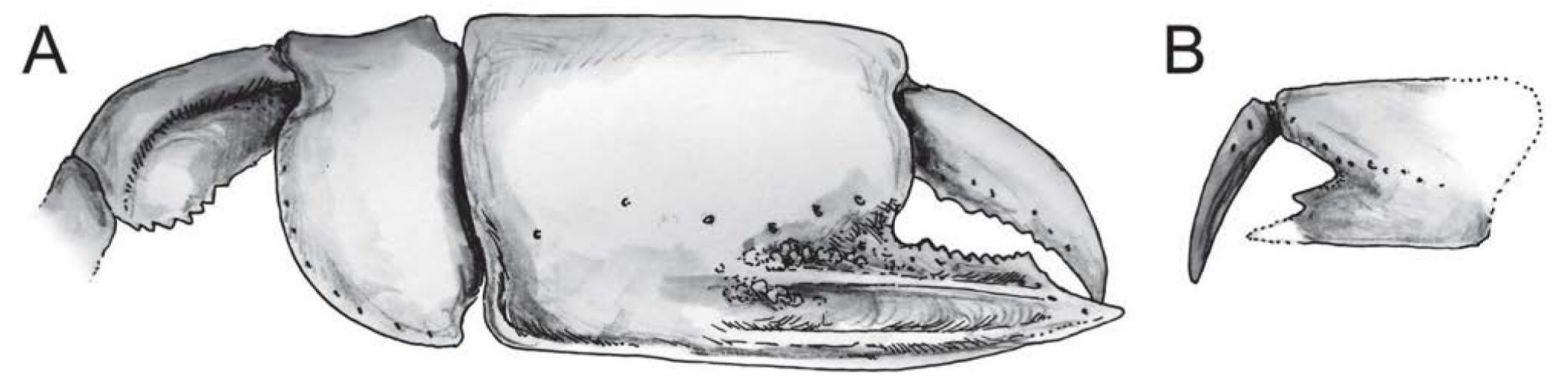
*Calliax michelottii* (A. Milne Edwards, 1860) comb. nov., reconstruction: A, major chela; B, minor chela (based on Fritsch 1871: pl. 17, fig. 14). Both chelae are depicted in outer lateral aspect.

The best preserved and most numerous remains of *Calliax* in the fossil record belong to species originally described as *Callianassa michelottii* (Figs 5–10). It is discussed in detail below.

Feldmann *et al.* (2005) reported several isolated cheliped elements from the Miocene of the Navidad Formation of Chile as Callianassoidea sp. 1. Although the authors stated that it does not resemble any callianassoid genus (Feldmann *et al.* 2005: 431), the figured material (Feldmann *et al.* 2005: fig. 2A) exhibits striking similarities with *Calliax* as discussed herein. Interestingly, *Callianassa szobensis* Müller, 1984, which is herein considered a junior subjective synonym of *C. michelottii* (see below) and hence a member of *Calliax*, is mentioned by Feldmann *et al.* (2005) as similar to their Callianassoidea sp. 1. The same locality also yielded another specimen which has been identified as *Callichirus* sp. The figured propodus (Feldmann *et al.* 2005: fig. 2A) shows a relatively short manus; however, the fixed finger with ridges and a furrow indicates its affinities to Callianassoidea sp. 1 (Feldmann *et al.* 2005: fig. 2D). Both specimens are treated here as *Calliax* sp. 1.

Feldmann *et al.* (2011) reported fragmented material from the Miocene of Tierra del Fuego (Argentina) as “Cheliped Form B” of indeterminate callianassoid. As already noted by Hyžný & Hudáčková (2012: 13), the minor chela exhibits remarkable similarities to *Calliax*, (Feldmann *et al.* 2011: fig. 5E) showing a fixed finger shorter than the dactylus and separated from it by a wide gap with a proximal tooth (cf. Ngoc-Ho 2003: 490). The major P1 propodus (Feldmann *et al.* 2011: fig. 5B), however, does not possess the ridges on the fixed finger. It is fairly likely that the two specimens do not belong to the same taxon, as they were not found associated with each other. For the purposes of this contribution only the minor chela is referred to here as *Calliax* sp. 2.

Charbonnier *et al.* (2013) reported a single near-complete propodus from the Paleocene of Pakistan identified as a minor chela of *Calliax*. Indeed, the specimen shows all features typical for minor chelae of the genus (Charbonnier *et al.* 2013: fig. 2) as discussed above. This occurrence is considered the oldest confirmed fossil record of the genus, treated here as *Calliax* sp. 3.

*Callianassa whiteavesi* Woodward, 1896 from the Campanian of Canada (Woodward 1896; Feldmann & McPherson 1980; Schweitzer *et al.* 2003) was assigned to *Calliax* by Schweitzer *et al.* (2003). The material is rich and sufficiently preserved to reconstruct both chelipeds (Feldmann & McPherson 1980). The species differs markedly from any *Calliax* species. It does not possess the typically shaped minor cheliped as discussed above, nor has it parallel ridges on the base of the fixed finger. Moreover, some specimens exhibit a rather deep dactylus, a character not observed in *Calliax*. As a result, the species is excluded from *Calliax* herein. Until the type material is restudied we suggest to keep the species under *Callianassa sensu lato*.

Swen *et al.* (2001) reported a single fragmentary right propodus from the Maastrichtian of the Netherlands as “*Calliax*? sp.”. The material is too fragmentary for resolving its generic status. The oblique development of the ridge at the base of fixed finger (Swen *et al.* 2001: fig. 5.3), however, points to closer affinities to *Eucalliax* or *Calliaxina* rather than *Calliax*.

Van Bakel *et al.* (2006) listed in a table of Cenozoic decapods from Belgium the presence of *Calliax* in the Miocene strata. The material was recently described as a new member of the family Axiidae (Fraaije *et al.* 2011).

#### Occurrence and distribution

Paleocene–Holocene. Two formally described extant species are known from West Atlantic (Florida) and Mediterranean (Sakai 2011). Based on the reports discussed above (Feldmann *et al.* 2005, 2011), the geographical distribution of the genus was much wider during the Miocene than today, and the genus was apparently also present in the East Pacific (see below). All occurrences are reviewed in Table 1.

*Calliax michelottii* (A. Milne Edwards, 1860) comb. nov.

(Figs 5A–D, 6A–L, 7A–M, 8A–L, 9A–I, 10A–B)

*Callianassa Michelotti* A. Milne Edwards, 1860: 341, pl. 14, fig. 3; A. Milne Edwards, 1860: 210, pl. 14, fig. 3.

*Callianassa michelotti*—Schweitzer *et al.*, 2010: 35.

*Callianassa Michelottii*—Fritsch, 1871: 691, pl. 17, figs. 5–13; Noetling, 1886: 84, pl. 5, fig. 4; Crema, 1895: 667, fig. 3; Glaessner, 1928: 167–168; Glaessner, 1929: 84.

*Callianassa michelotii*—Beurlen, 1931: 111–112.

“*Callianassa*” *michelotti*—Polkowsky, 2005: 17, figs. 8–13, pl. 1, figs. 1–8.

*Callianassa* cf. *michelottii*—Moths & Montag, 2002: 7, pl. 4, fig. 3.

‘*Callianassa*’ *szobensis* Müller, 1984: 53 (partim), pl. 7, figs.3–4.

*Callianassa szobensis*—Schweitzer *et al.*, 2010, 37.

“*Callianassa*” *szobensis*—Hyžný, 2011: table 2.

*Callianassa* sp.—Houša *in* Špinar *et al.*, 1965: 734, figs. X-184–185.

? *Calianassa Michelotti*—Wagner-Klett, 1919: 107, pl. 2, fig. 1.

? ‘*Callianassa*’ *szobensis* Müller, 1984: 53 (partim); pl. 7, figs. 5–6.

? *Callianassa* sp.—Philippe & Secretan, 1971: 128, pl. C, figs. 13–14.

? Eine nicht bestimmbare Hand—Lőrenthey, 1907: 212, 222, pl. 1, fig. 5.

non *Callianassa* cf. *michelottii* Müller, 1993: 7, fig. 3A.

#### Diagnosis

Major cheliped massive; merus longer than high (L/H=2.2) with convex upper margin, carpus higher than long (L/H=0.45), about half the length of manus or shorter, upper margin straight, proximolower margin regularly rounded and smooth in outline; propodus rectangular, longer than high, exhibiting two morphotypes (L/H=1.1–1.2; 1.3–1.5); outer lateral surface of manus smooth, adorned with several tubercles; fixed finger shorter than manus, triangular, with two parallel ridges extending onto manus, lower (less developed) ridge positioned close to the lower propodal margin; occlusal margin of fixed finger serrated and adorned with a blunt tooth pointing up and forward; dactylus slender, with serrated occlusal margin and pointed tip.

#### Description

Major cheliped massive. Merus longer than high (L/H=2.2), with prominent keel along the midline; upper margin unarmed, convex; lower margin concave, serrated. Carpus higher than long (L/H=0.45), upper margin straight or concave, proximolower margin regularly rounded and smooth in outline. Propodus rectangular, longer than high (L/H=1.1–1.5), distinctly longer than carpus; upper and lower margins straight, parallel to each other, forming distinct keels on the inner sides, rounded proximally; distal margin straight, parallel to proximal margin, the base of the fixed finger may form a notch. Outer lateral surface of manus smooth, adorned with several tubercles (at the bases of setal pores) lying in a row positioned obliquely. Inner lateral surface of manus concave, with distinct depression at the base of fixed finger. Fixed finger shorter than manus, triangular, with two ridges extending onto manus, lower (less developed) ridge positioned close to the lower propodal margin, upper one (strongly developed) accompanied with several tubercles proximally; occlusal margin serrated and adorned with a blunt tooth pointing up and forward. Dactylus slender, with serrated occlusal margin and pointed tip.

#### Variations

Major propodi of *Calliax michelottii* comb. nov. exhibit a certain degree of variation. When examining a large number of specimens, one can observe differences in the manus length/height ratio attaining values in two intervals, i.e. 1.1–1.2 (shorter morph) and 1.3–1.5 (longer morph). Unfortunately it is difficult to state whether it is intraspecific variation or if it mirrors sexual dimorphism. Polkowsky (2005) tentatively interpreted two morphotypes of *C. michelottii* as sexual dimorphs. Some propodi may possess a relatively well developed notch at the base of the fixed finger. Its development seems to be at least partly correlated with the size, but as already pointed out by Polkowsky (2005), the notch in the longer morphotype is usually better developed. Development of the ridges is a stable character, i.e. there are always two ridges present, and the lower one is rather faint. What differs in various propodi is the shape of the upper ridge in its proximalmost part. In some specimens it is straight, but in others it is curved downwards. The development of tubercles accompanying the upper ridge also varies, depending on the size of the animal.

Glaessner (1928) reported strong ornamentation on the isolated propodi from Jarenina, Slovenia (Figs 8A–B). This can, however, be ascribed mainly to preservational aspects. Specimens from Želiezovce, Slovakia (Špinar *et al.* 1965: fig. X-184; refigured here as Fig. 8L) and Szob, Hungary (Müller 1984: pl. 7, figs. 3, 4; refigured here as Figs 8J–K) exhibit a similar pattern of ornamentation, although not so pronounced as in the material from Jarenina. Possibly it is related to calcification of the cuticle. Experimental data, which are lacking at this point of time, would help in elucidating this issue.

Major P1carpus may have a concave upper margin (Fritsch 1871: pl. 17, fig. 9; refigured here as Fig. 6E). A similarly concave upper margin was also observed in extant *Calliax doerjesti* (Sakai 1999: fig. 28, 29; Fig. 3A). This feature may be related to age and/or size of the individual. For resolving this issue in *Calliax michelottii* comb. nov. more preserved carpi must be examined.

#### Material examined

Only remains of major chelipeds have been examined: the holotype of *Callianassa michelottii* from Superga at Turin, Italy (MNHN-F-B32690; Fig. 5); 21 fragmentary propodi and one carpus (NHMW 1874/0029/1154 a–v) from Sternberg, Germany (Fig. 7); one isolated propodus from Pinnow bei Schwerin, Germany (NHMW 1874/0029/1155; Fig. 7I); one right propodus articulated with dactylus from Pucking, Austria (NHMW 2003/0026/0913; Fig. 8C); entire articulated right cheliped from Neuhofen bei Tettenweis (NHMW 2010/0089/0001; Fig. 8I); two isolated left propodi (RGA/SMNH 0773, 0779), two isolated right propodi (RGA/SMNH 0754, 0864), four articulated chelae consisting of propodus and dactylus (RGA/SMNH 1075, 1187) and even carpus and merus (RGA/SMNH 1069, 1191) from Kamnik-Košiše, Slovenia (Figs 9A–H); right isolated propodus from the unknown site at Kamnik (Stein in Krain), Slovenia (GBA 2009/014/0027; Fig. 9I); two isolated propodi from Jarenina (Jahring), Slovenia already reported (but not figured) by Glaessner (1928) (UMJGP 77873–77874; Figs 8A–B); one right fragmentary propodus from Rohožník, Slovakia (KGP-MH RO-001; Figs 8D–H); holotype of *Callianassa szobensis* from Szob, Hungary (M.2004.158.1; Figs 8J–K).

#### Occurrence

Oligocene (Rupelian)–Middle Miocene (Serravallian) of Europe. The oldest occurrence of the species is known from the Rupelian of the Mainz Basin, Germany (Fritsch 1871; Figs 6A–G). The youngest one is from the Middle–Late Badenian (Serravallian) of Slovakia (herein). All confirmed occurrences are summarized in Table 2. For details see text below.

#### Remarks

*Callianassa michelottii* was originally described based on isolated propodi (Fig. 5) from the Miocene of Superga hill near Turin, Italy (A. Milne Edwards 1860). A. Milne Edwards (1860: p. 211) noted that the presence of the keel on the fixed finger accompanied by the tubercles above it is unique not only for fossil species, but this character distinguishes the species from all extant callianassids known at that time as well. Indeed, the type species of *Calliax* was described more than a century later by de Gaillande & Lagardère (1966). Crema (1895) reported *C. michelottii* from the Middle Miocene of nearby localities in the Turin hills.

Fritsch (1871) reported *C. michelottii* from the “Middle” Oligocene (Rupelian) “Septarienthon” of the Mainz Basin (W Germany). Interestingly, Fritsch (1871: pl. 17, figs 5–13) reported and also figured a small chela preserved together with the major P1 propodus (Fig. 6G). He tentatively interpreted it as remains of P2, but it in fact represents a minor P1 chela. The description of Fritsch (1871: 696) is very clear in this sense (“Der bewegliche Finger steht auf einem weit nach vorn vorspringenden Theil des Ballens. Vom unbeweglichen Finger ist nu rein ganz geringer Theil sichtbar, warscheinlich war derselbe indess kurz, die hervortretenden Leisten desselben Gliedes der Vorderhand fehlen.” English translation: Dactylus is located on the manus portion projecting forward. Only very small portion of the index is visible. It was probably short, although the tip is missing.) Fritsch (1871: 692) mentioned also the presence of the cephalothorax (“Wohl liegt ein Exemplar vor, an welchem man Theile des kleinen dünnschaligen Cephalothorax und der seitlich zusammengedrückten hinteren Fusspaare erkennt, doch is dasselbe für eine Charakteristik des Thieres zu ungenügend erhalten.” English translation: There is a sample in which the individual shows the preserved small cephalothorax with thin cuticle and the laterally compressed “rear” pereiopod. However, the preservation of both characters is insufficient.), but he did not figure it. The material of Fritsch (1871) was reexamined by Beurlen (1931); he confirmed much of what was already done in the original work.

Noetling (1886: pl. 5, fig. 4; refigured here as Figs 6H–K) reported the species from the Late Oligocene “Sternberger Gestein”of Kobrow, Germany. The latest contribution on *Callianassa michelottii* from this facies is that by Polkowsky (2005) who discussed at length the variation of the species as well as its geographic distribution.

Lőrenthey (1907: pl. 1, fig. 5; refigured in Fig. 6L) reported and figured undetermined isolated propodi from the Miocene of Cagliari, Sardinia. With two parallel ridges on the fixed finger, the specimen shows affinities to *Calliax michelottii* comb. nov., but possesses rather convex upper propodal margin, which is unusual for *Calliax*, although the character itself may reflect mere intraspecific variation. The material (collection of Lovisato) is considered lost (De Angeli & Garassino 2006); thus, the re-examination of the material is not possible. As a consequence, we treat the occurrence as questionable.

Wagner-Klett (1919) reported *Callianassa michelottii* from the Oligocene Septarienton of Wiesloch, Germany. The figure he provided (Wagner-Klett 1919: pl. 2, fig. 1), however, does not conform to the diagnosis of the species. The specimen consisting of an articulated carpus, propodus, and dactylus shows no ridges on the fixed finger. The material was either not properly figured or it represents a different species.

Glaessner (1928) reported the species from the Helvetian (Early Miocene) of Jahring (today’s Jarenina, Slovenia) in the Styrian Basin. He, however, did not provide figures. The re-examination of the material by the senior author confirms the identification of the material (Figs 8A–B).

Beurlen (1939: 143) when discussing affinities of *Callianassa brevimanus* Beurlen, 1939, (currently recognized as a member of *Lepidophthalmus* Holmes, 1904; see Hyžný & Dulai in press) briefly mentioned *C. michelottii* as coming from the Oligocene of the Mainz Basin and Miocene of Italy and the Vienna Basin, however, without any further reference. It is supposed here that Beurlen (1939) mentioned the occurrence from the Vienna Basin erroneously, as there is no such published report known to the authors. He might have been referring to the published occurrence from the Styrian Basin by Glaessner (1928).

Several chelae from the Middle Miocene (“Badenian”) of Želiezovce (Slovak part of the Danube Basin) figured by Houša *in* Špinar *et al.* (1965: figs. X-184–185, refigured herein as 8L) as *Callianassa* sp. can be clearly assigned to *Callianassa michelottii*. Unfortunately, the repeated search for the material was unsuccessful, thus, it is herein considered lost.

Philippe & Secretan (1971) reported 10 fragmentary propodi from the Burdigalian of SE France. The figured specimens (Pl. C, figs. 13, 14) show the keeled fixed finger accompanied with furrows, thus, pointing to attribution of the material to *Calliax*. The age and location of the specimens would speak for indentification as *C. michelottii*. Without personal re-examination of the material, however, we are reluctant to treat the specimens as conspecific with *C. michelottii*.

The description and figures of “*Callianassa*” *szobensis* Müller, 1984 from the Middle Miocene (Badenian) of Hungary fit the diagnosis and variations of *C. michelottii*. The propodus is longer than high, the fixed finger possesses two ridges parallel to one another, and there are tubercles on the lateral surface of the propodus (Figs 8J–K). As a consequence, “*C.*” *szobensis* is considered a junior subjective synonym of *C. michelottii*.

From the Miocene of Spain (Catalonia), Müller (1993: fig. 3A) reported and figured an isolated propodus of a callianassid shrimp. He classified it as *Callianassa* cf. *michelottii*. The specimen, however, is rather dissimilar to *C. michelottii*; it does not possess a double ridge on the fixed finger and has the upper margin converging proximally, which is very atypical for the latter species.

*Calliax michelottii* n. comb. is morphologically very close to its extant congeners. The upper margin of the major P1 merus is, however, more convex in *C. michelottii* n. comb. (Fritsch 1871: pl. 17, fig. 9; see also Figs. 8I), whereas it is rather straight in both extant species, *C. doerjesti* and *C. lobata* (Fig. 3A and Fig. 3C, respectively). The development of spines on the lower margin of the major P1 merus in *Calliax michelottii* comb. nov. is closer to that of *C. lobata*. The tooth formula of the occlusal margin of the fixed finger looks different in all three species, but this may be a matter of variation and is not considered taxonomically important here.

### Notes on preservation

Mostly only isolated major P1 propodi of *Calliax michelottii* comb. nov. have been recovered (A. Milne Edwards 1860; Fritsch 1871; Noetling 1886; Polkowsky 2005). There are several basic types of preservation. When preserved three-dimensionally, the cuticle often is shiny and smooth (e.g. Fritsch 1871; Polkowsky 2005).

Reports on articulated individuals are scarce and are limited to preservation in fine siliciclastics (Figs 8I, 9E, 9H).

The scarcity of major P1 carpi and meri as well as of minor P1 elements can be ascribed to rather low fossilization potential of these elements as a consequence of their weak calcification. Another factor influencing our knowledge in a negative way may be collecting and reporting bias, as smaller pieces often are more difficult to interpret and may be neglected by collectors donating the material to scholars.

Interestingly, *Calliax michelottii* comb. nov. has been reported associated with *Ophiomorpha* Lundgren, 1891, burrows (Polkowsky 2005). Herein, another occurrence is reported, specifically from Košiše outcrop, where isolated propodi have been found in direct proximity of burrow structures. Preservation of the ghost shrimp body fossils *in situ* within their burrows or in direct association with them are often considered scarce (Bishop & Williams 2005; Hyžný 2011); thus, every report in this respect is of note.

### Palaeoenvironmental significance

Today *Calliax* seems to inhabit a wide range of habitats. *Calliax lobata* is known from very shallow water environments up to 21 m (Ngoc-Ho 2003), whereas *Calliax* sp. reported by Taviani *et al.* (2013) has been found at ca. 800 m depth. Unfortunately, the bathymetry is not known for *Calliax doerjesti* (Sakai 1999, 2011).

Taviani *et al.* (2013) reported great densities of *Calliax* sp. in the pockmark mud of the strait of Sicily around a cold seepage, where an assemblage of chemosymbiotic organisms has been recovered. In the environment, carbonate concretions were formed (Taviani *et al.* 2013). Such sedimentological conditions can be compared to the situation in the Middle Miocene locality at Rohožník, Slovakia, where carbonate concretions contain mollusc shells together with remains of fish and decapod crustaceans, including *Calliax michelottii* comb. nov. No closer examination of the concretions or faunal elements has been conducted so far, thus the analogy is pure speculation at this moment. The preliminary results based on foraminifers and molluscs, however, point to deeper marine conditions with intervals of shallow littoral sea influx (Fuksi *et al.* 2011).

The holotype of *Callianassa michelottii* was recovered from the “Serpetinensand”. A. Milne Edwards (1860), however, supplied little data on the sedimentological conditions and no further speculations can be made. In the Oligocene of Germany, *Calliax michelottii* comb. nov. is known mainly from the so called “Septarienton” (=Rupelton, i.e. clay with concretions) (Fritsch 1871) and “Sternberger Gestein”, i.e. Sternberg erratic boulders of Mecklenburg (Noetling 1886; Polkowsky 2005). From the latter facies, great densities of *C. michelottii* are known (Polkowsky 2005). At the locality of Kobrow, *Ctenocheles rupeliensis* (Beurlen, 1939) also has been found (Moths & Montag 2002; Polkowsky 2005; Hyžný & Dulai in press), which is considered to be an inhabitant of deep-water settings (Hyžný & Dulai in press, and references therein).

Glaessner (1928) reported *C. michelottii* from the “Steirischer Schlier” (clays of the Kreuzkrumpel Formation), which was presumably deposited at the depth between 225 m and 315 m (Hohenegger *et al.* 2009 and references therein).

Considering *Calliax michelottii* comb. nov. an indicator of deep water conditions may be premature; so far, however, its remains are almost unequivocally connected with facies deposited in such conditions.

### Notes on palaeobiogeography

The oldest fossil record of *Calliax* as recognized herein is that from the Late Paleocene of Pakistan (Charbonnier *et al.* 2013); thus, it seems the genus has a Tethyan origin. Unfortunately, so far no Eocene occurrences are known; therefore, it is rather difficult to interpret the dispersal pattern of the genus. Only a few remarks can be made.

**FIGURE 11:**
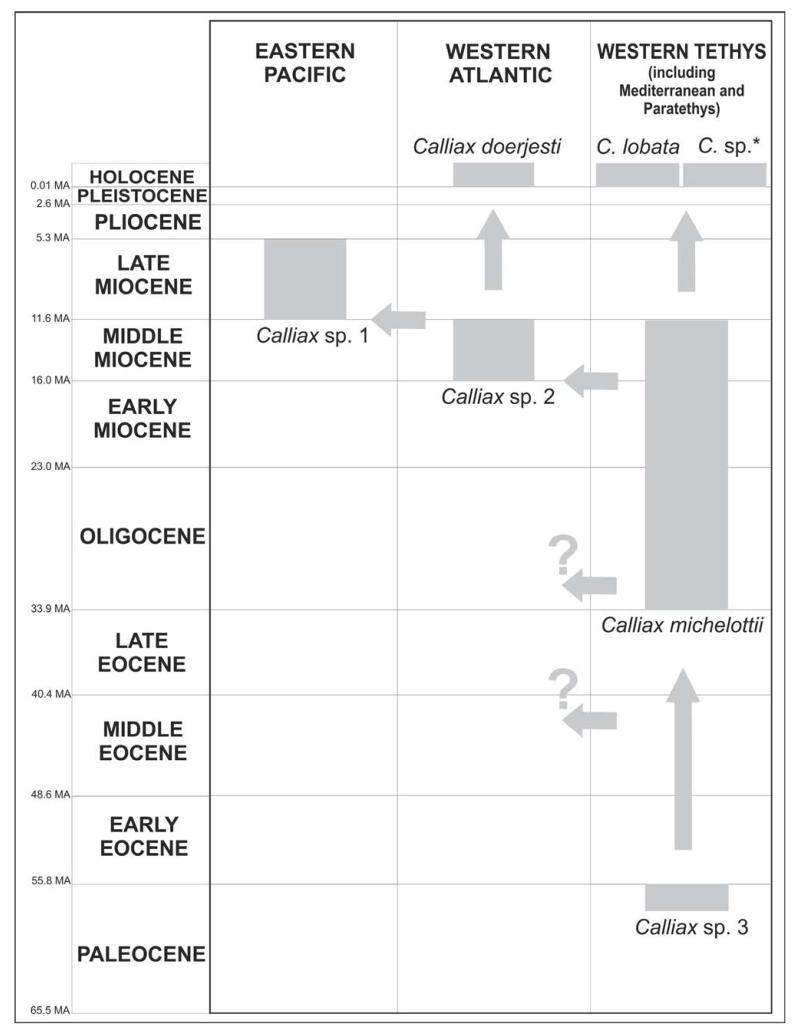
Biogeographical evolution of *Calliax*. Chronostratigraphical dates are adopted from Gradstein *et al.* (2004). C. sp.* =*sensu* Taviani *et al.* (2013)

Today, *Calliax* has been reported only from the Mediterranean and Atlantic Ocean (Sakai 2011). The fossil record shows that the genus was present in the Proto-Mediterranean and Paratethys seas during Oligocene and Miocene times. Thus, *Calliax* has occupied the Western Tethys at least since the Paleocene (Fig. 11). It remains to be answered when the dispersal across the Atlantic Ocean actually took place, as the genus is known from the Miocene of Argentina; at least the minor chela reported by Feldmann *et al.* (2011) suggests it. The confirmed record from the Late Miocene of Chile (Feldmann *et al.* 2005), i.e. Eastern Pacific, may support the previous occurrence. The pattern of supposed oceanic circulation during the Cenozoic as presented by Feldmann & Schweitzer (2006: fig. 3), however, suggests that *Calliax* dispersed into the Eastern Pacific *via* the Proto-Caribbean area and later reached the southernmost part of South American continent. More fossil occurrences are needed to further speculate on this issue. A westward migratory route across the Atlantic Ocean during the Oligo-Miocene time has been suggested for several marine groups, including ghost shrimps (Harzhauser *et al.* 2002, 2007; Hyžný & Müller 2012).

## Conclusions

1. Based on thorough comparison between extant and fossil ghost shrimps, a set of characters present on P1 are considered of taxonomic importance at the genus level. The combination of rectangular major P1 propodus with short fixed finger exhibiting two more-or-less parallel ridges extending onto the manus and a minor P1 with the fixed finger distinctly shorter than the dactylus and with a wide gap between the fingers is unique among ghost shrimps and characterizes *Calliax*. Even the morphology of the major P1 propodus alone is distinct enough to be safely assigned to *Calliax*, yet the fossil specimens have been rarely interpreted as members of this genus.
2. *Callianassa michelottii* originally described from the Miocene of NW Italy (A. Milne Edwards 1860) and later widely reported from the Oligocene and Miocene of Europe (Fig. 1, Table 2) is re-assigned to *Calliax*. *Callianassa szobensis* from the Middle Miocene of Hungary (Müller 1984) is considered a junior subjective synonym of *C. michelottii*. New occurrences of the species are reported from the Neogene basins of the former Central Paratethys Sea, specifically from the North Alpine Foreland Basin and the Vienna Basin.
3. The presence of *Calliax michelotti* comb. nov. may be considered an indicator of deeper marine settings. This hypothesis, however, must be further tested.
4. The fossil record of *Calliax* is revised and the presence of the genus in the Miocene strata of South America is documented. Compared to today’s occurrences, the geographic distribution of *Calliax* was wider in the geologic past. Based on the fossil record known so far, a Tethyan origin for the genus is postulated.

## Figures and Tables

**TABLE 1:** Synopsis of fossil and extant occurrences of *Calliax* de Saint Laurent, 1973 known to date.

Taxon	Age	Distribution	Reference
Species with an exclusively recent record			
*Calliax doerjesti*		Florida	Sakai (1999)
*Calliax lobata*		Mediterranean	Ngoc-Ho (2003)
*Calliax* sp. *sensu* Taviani *et al.* (2013)		Mediterranean (Strait of Sicily)	Taviani *et al.* (2013)
Fossil occurrences			
*Calliax* sp. 1	Late Miocene	Chile	Feldmann *et al.* (2005)
*Calliax* sp. 2	Middle Miocene	Argentina	Feldmann *et al.* (2011)
*Calliax michelottii* **n. comb.**	Early Oligocene– Middle Miocene	Europe	herein
*Calliax* sp. 3	Late Paleocene	Pakistan	Charbonnier *et al.* (2013)

**TABLE 2:** Synopsis of confirmed occurrences of *Calliax michelottii* (A. Milne Edwards, 1860) comb. nov. The occurrences are arranged according to their stratigraphic age.

Locality	Age	Reference
Flörsheim (D)	Early Oligocene	Fritsch (1871), Beurlen (1931)
Offenbach bei Frankfurt (D)	Early Oligocene	Fritsch (1871)
Sternberg (D)	Late Oligocene	herein
Pinnow bei Schwerin (D)	Late Oligocene	herein
Kobrow (D)	Late Oligocene	Noetling (1886), Polkowsky (2005)
Pucking (A)	Early Miocene (Late Egerian)	herein
Neuhofen (D)	Early Miocene (Late Ottnangian)	herein
Jarenina (SLO)	Early Miocene (Karpatian)	Glaessner (1928)
Szob (H)	Middle Miocene (Early Badenian)	Müller (1984)
Rohožník (SVK)	Middle Miocene (Late Badenian)	herein
Kamnik, Košiše (SLO)	Middle Miocene (Badenian)	herein
Želiezovce (SVK)	Middle Miocene (Badenian)	Špinar et al. (1965), herein
Superga, Turin hills (I)	Middle Miocene	A. Milne Edwards (1860), Crema 1895
